# Athletes Can Benefit from Increased Intake of EPA and DHA—Evaluating the Evidence

**DOI:** 10.3390/nu15234925

**Published:** 2023-11-26

**Authors:** Maja Tomczyk, Jeffery L. Heileson, Mirosław Babiarz, Philip C. Calder

**Affiliations:** 1 Department of Biochemistry, Gdansk University of Physical Education and Sport, 80-336 Gdansk, Poland; 2 Department of Health, Human Performance, and Recreation, Baylor University, Waco, TX 76706, USA; 3 Nutrition Services Department, Walter Reed National Military Medical Center, Bethesda, MD 20814, USA; 4 Department of Physiology, Gdansk University of Physical Education and Sport, 80-336 Gdansk, Poland; mirekbabiarz@scec.pl; 5 School of Human Development and Health, Faculty of Medicine, University of Southampton, Southampton SO16 6YD, UK; p.c.calder@soton.ac.uk; 6 NIHR Southampton Biomedical Research Centre, University Hospital Southampton NHS Foundation Trust and University of Southampton, Southampton SO16 6YD, UK

**Keywords:** omega-3 fatty acids, eicosapentaenoic acid, docosahexaenoic acid, supplementation, athletes

## Abstract

Fatty fish, which include mackerel, herring, salmon and sardines, and certain species of algae (e.g., *Schizochytrium* sp., *Crytthecodiniumcohnii* and *Phaeodactylumtricornutum*) are the only naturally rich sources of the omega-3 polyunsaturated fatty acids (*n*-3 PUFAs) eicosapentaenoic acid (EPA) and docosahexaenoic acid (DHA). EPA and DHA are the most biologically active members of the *n*-3 PUFA family. Limited dietary sources and fluctuating content of EPA and DHA in fish raise concerns about the status of EPA and DHA among athletes, as confirmed in a number of studies. The beneficial effects of EPA and DHA include controlling inflammation, supporting nervous system function, maintaining muscle mass after injury and improving training adaptation. Due to their inadequate intake and beneficial health-promoting effects, athletes might wish to consider using supplements that provide EPA and DHA. Here, we provide an overview of the effects of EPA and DHA that are relevant to athletes and discuss the pros and cons of supplements as a source of EPA and DHA for athletes.

## 1. Introduction

Athletes might consider using supplements for a variety of reasons. Any supplement should be safe and the decision to use it should be based on an evaluation of the evidence in its favour and of any possible adverse impacts. Having guidance around supplement use is valuable to athletes, coaches and support staff. One of the most reliable classifications of dietary supplements used by athletes is the Australian Institute of Sport (AIS) Framework [1]. The AIS classifies dietary supplements based on scientific evidence, starting with A—whose scientific support is the strongest; through B—in which a scientific background appears but deserves further research; and C, in which existing scientific evidence does not support benefits among athletes OR no research has been conducted to reach a clear conclusion. Finally, category D includes dietary supplements banned by the World Anti-Doping Agency, the detection of which in the body of an athlete could lead to their exclusion from sports competition. Category B includes fish oil, a source of the *n*-3 polyunsaturated fatty acids (PUFAs) eicosapentaenoic acid (EPA) and docosahexaenoic acid (DHA) [1]. This categorization indicates that there is a supportive scientific background for EPA and DHA, but that further research is needed.

**Figure 1:**
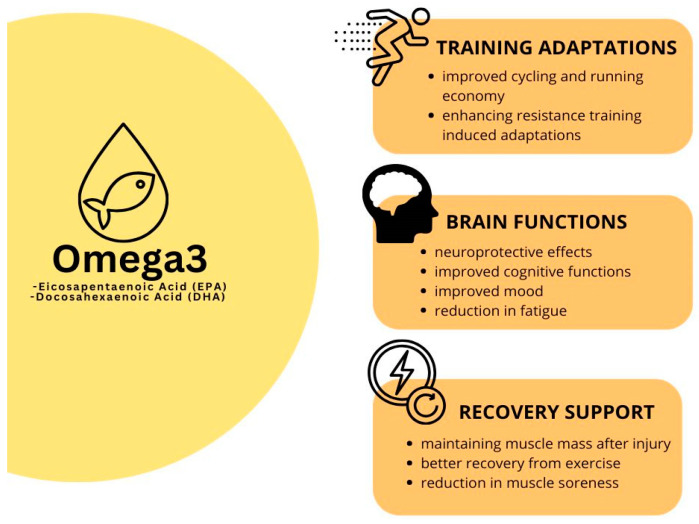
The most important effects of EPA and DHA in athletes based on the current state of knowledge.

The defining feature of all *n*-3 PUFAs is the presence of the first double bond in the fatty acyl chain being on the third carbon atom from the methyl end of the chain. The family of *n*-3 PUFAs includes alpha-linolenic acid (ALA), EPA and DHA. The human body lacks the ability to insert a double bond in the “omega-3” position of the fatty acyl chain and therefore *n*-3 PUFAs cannot be produced de novo and must be obtained from the diet or through supplementation [2]. Plant foods such as nuts, flaxseeds and chia seeds do not contain the most biologically active *n*-3 PUFAs, EPA and DHA, which are only found in high amounts in oily fish and selected species of algae [2,3]. The precursor *n*-3 PUFA is ALA, which is found in plant sources (green leaves, seeds, nuts, some vegetable oils). ALA may undergo metabolic conversion by enzymatic transformations to form the longer-chain more unsaturated EPA and DHA. However, based on stable isotope tracer studies, such conversion is significantly limited [4,5,6,7,8], with one study estimating enzymatic conversion at 0.2% to EPA and 0.05% to DHA [9]. Because of this limited conversion of ALA, the intake of preformed EPA and DHA is important in maintaining EPA and DHA status. The best marker reflecting the intake and status of EPA and DHA is the so-called omega-3 index (O3I), that is, the sum of EPA and DHA expressed as a percent of total fatty acids in erythrocytes, as proposed by Harris and von Schacky [10]. O3I values that are considered to be optimal for the general population are >8%, while values between 4 and 8% and below 4% are considered average and insufficient, respectively [10]. These cut-offs are based mainly on cardiovascular disease outcomes [10]. Low O3I is common in many populations, mainly reflecting a low intake of fatty fish. Existing studies indicate that both professional and amateur athletes have O3I values below optimal [11,12,13,14,15,16,17]. In this regard, athletes are most likely reflective of the general population. For example, Arsic et al. [18] reported that the O3I of female elite athletes (water polo and football players) was low but not different from that of sedentary women from the same population. Considering this low O3I, as well as the scarcity of EPA and DHA in foods and their pleiotropic effects, including but not limited to the control of inflammation, support of cognitive function, neuroprotection, maintaining muscle mass after injury and enhancement of training adaptations and recovery from exercise [19,20,21,22,23,24,25,26,27], the question arises about whether athletes should consider using supplements that contain EPA and DHA. The main goal of this article is to provide an overview of the principal effects of EPA and DHA that are relevant to athletes (Figure 1) and to discuss their pros and cons.

## 2. Effects of EPA and DHA and Underlying Mechanisms

Human metabolism and physiology are rife with examples of molecular structure influencing function. Indeed, EPA and DHA, by virtue of their long carbon chain length, high degree of unsaturation and presence of a double bond next to the third carbon atom from the methyl end, have unique physical and biochemical properties. They sometimes act through cell surface G-protein coupled receptors such as GPR120, they influence the activation of important transcription factors including the peroxisome proliferator activated receptors (PPARs) and nuclear factor kappa-light-chain-enhancer of activated B cells (NFκB) and they directly influence cell membrane structure and function through incorporation into phospholipids; these mechanisms of action are reviewed elsewhere [28,29]. Many of these mechanisms of action are interacting. This is perhaps most evident for the anti-inflammatory effects of EPA and DHA, which involve (a) decreased production of bioactive oxylipins from the *n*-6 PUFA arachidonic acid (e.g., prostaglandin D_2_ and E_2_, 4-series leukotrienes) due to the displacement of arachidonic acid from cell membrane phospholipids and (b) inhibition of the activation of NFκB and subsequent reduced expression of NFκB-target genes encoding a range of proteins involved in inflammation, including tumour necrosis factor (TNF)-alpha, interleukin (IL)-6, IL-1β and cyclooxygenase-2, as reviewed elsewhere [30]. The inhibition of NFκB activation appears to involve several sites of action. GPR120 is a cell surface receptor for which DHA is a good ligand [31]. GPR120 signalling results in the inhibition of NFκB, and this mechanism was demonstrated to be important in mediating the anti-inflammatory effects of DHA in macrophages and insulin-sensitising effects in adipocytes, as reviewed elsewhere [32,33]. The inhibition of NFκB activation by DHA [34,35] has also been linked to the suppressed formation of membrane lipid rafts as shown in macrophages and dendritic cells [36,37]. Finally, EPA and DHA are ligands for, and activators of, PPARs [38,39], and PPARs physically interact with NFκB preventing its translocation to the nucleus [40]. EPA and DHA also act through conversion to bioactive mediators. These include a range of oxylipins (i.e., oxidised EPA and DHA derivatives) with biological activity including a range of hydroxy-EPAs and -DHAs [41,42]. Amongst these oxylipins are families of molecules now known to be central players in the resolution of inflammation. Collectively, these are termed specialized pro-resolving mediators (SPMs) and they include resolvins, protectins and maresins [42,43]. SPMs have widespread actions in supporting immunity, promoting the clearance of cellular debris, terminating inflammation, inducing wound healing and tissue repair and establishing homeostasis [42,43]. Clearly such actions are all relevant to the athlete. The conversion of EPA and DHA to SPMs and other oxylipins is now considered to be a major mechanism underpinning their action. Given this, suboptimal dietary and, thus, blood levels of EPA and DHA may reduce the body’s efficiency to resolve inflammation, consequently adversely affecting the progression and severity of various diseases that involve inflammation. These include both classic inflammatory diseases like arthritis, inflammatory bowel diseases and asthma [44,45] but also chronic conditions of ageing such as atherosclerosis, diabetes, cognitive decline and the loss of lean body mass [46,47]. Beyond oxylipins, EPA and DHA are components of other bioactive molecules including endocannabinoids that have roles in regulating appetite, inflammation and cognition [48,49,50,51,52].

DHA plays a crucial role in the nervous system. More than 50% of the dry weight of the human brain is lipid, of which DHA comprises 10–20% of the total pool and 90% of the *n*-3 PUFAs; DHA is abundant in neuronal cell membranes and brain grey matter. DHA is essential for nerve impulse transmission, neuronal membrane stability, neuroplasticity and cell communication [53,54]. Therefore, DHA may have a neurosupportive role in the context of sport-related traumatic brain injury, cognitive function or even neuromuscular function associated with improvements in muscle strength [20,21,22,23]. One of the hypotheses for improved resistance exercise training (RET)-induced adaptations in skeletal muscle after long-term supplementation of EPA and DHA is fast-twitch muscle fibre hypertrophy [22]. Given the fact that the fast-twitch muscle fibre cross-sectional area (fCSA) has not been studied thus far, this provides a window of opportunity for future research. Finally, through stimulating the process of myofibrillar protein synthesis [55] which may be impaired for example due to leg immobilization, EPA and DHA supplementation may promote the preservation of muscle mass during a period of recovery from injury [25].

## 3. Nutritional Considerations about EPA and DHA

In contrast to ALA, which is produced in plants and consequently is present in plant-based foods such as seeds, nuts, green leaves and vegetable oils, EPA and DHA are present in significant amounts only in oily fish and some species of algae. The fish most abundant in EPA and DHA are mackerel and salmon (~1.8 g per 100 g), sardines (~1.6 g per 100 g), herring (~1.2 g per 100 g) and trout (~1.0 g per 100 g); they are present in lower amounts in cod (~0.24 g per 100 g) and other white fish [56]. However, there are at least three important factors that may contribute to the insufficient consumption of EPA and DHA from fish. Their content in fish is highly variable and depends on the metabolic profile of the fish, their maturity, the living conditions and food they ate/are fed and the season of the catch [57]. For example, one portion of salmon or mackerel could supply between 1.5 and 3.0 g of these fatty acids depending upon the above-mentioned conditions. In addition, there is evidence that the taste and smell of fish is one of the main reasons for their low consumption in the population, including among athletes [58]. Finally, there are health concerns regarding the possible presence of heavy metals in fish and their detrimental effects, which may affect the frequency of their consumption. In addition to EPA and DHA, fish are a source of high-quality protein and well-absorbed minerals and vitamins. It therefore appears that the health benefits of eating fish exceed the risks [59].

Species of microalgae, such as *Schizochytrium* spp., *Crytthecodiniumcohnii* and *Phaeodactylumtricornutum* are plant sources of EPA and DHA that can be used for producing supplements. For example, the oil content of *Schizochytrium* spp. can reach above 50% of the dry weight, while DHA levels can be over 35% of the total fatty acid content [60,61]. Currently, there is no dietary reference intake (DRI) for EPA and DHA, only adequate intake (AI) or dietary guidelines for the general population set by specific authorities or international associations. The AI for the sum of EPA + DHA according to the European Food Safety Authority (EFSA) is 250 mg/day for adults [62], which is in line with the recommendations of the Food and Agriculture Organization (FAO)/World Health Organization (WHO) [63] and Dietary Guidelines for Americans [64]. Intakes above 500 mg/d in total are recommended by the Academy of Nutrition and Dietetics and the International Society for the Study of Fatty Acids and Lipids [65,66]. Although two servings of oily fish per week provide the recommended intake of EPA and DHA, there are data indicating that this amount does not raise the O3I ≥ 8% [67,68]. A study by Jackson et al. evaluating the association of declared fish consumption and supplement intake with O3I in 3458 adults showed that those consuming fish twice per week had an O3I < 6 [67]. Moreover, in order to achieve an O3I > 8, one would need to consume fish three times per week and additionally take EPA + DHA supplements [67]. In support of this, Block et al. estimated that even individuals consuming at least two servings of fish per week and taking a supplement did not achieve an O3I > 8 [68]. Thus, the optimal intake of EPA + DHA (i.e., the amount needed to reach an O3I ≥ 8%) may be well above current dietary guidelines [69]. In a recent scoping review, Dempsey et al. evaluated 58 studies assessing the impact of EPA and DHA intake on O3I. They observed that the lowest doses that were effective in elevating O3I to recommended values were >1000 mg of a combination of DHA + EPA or DHA only daily, for periods of 12 weeks or longer [69]. Based on the data analysed, it was also suggested that in order to improve O3I to ≥ 8%, 1000–1500 mg/day of EPA + DHA for at least 12 weeks would be needed, while individuals with baseline O3I levels < 4% are likely to require longer durations of supplementation and/or higher dosages [69]. Currently, there are no specific recommendations for EPA and DHA intake and the duration of supplementation in the athlete population. A summary of the scientific evidence for the application of popular dietary supplements, published in a statement by the International Olympic Committee (IOC), indicates a dose of about 2 g of EPA + DHA daily which may offer potential health and performance effects [70].

## 4. Status of EPA and DHA in Athletes

Most of the studies conducted on athletes have indicated that O3I values are commonly below the optimum established for the general population, i.e., < 8%, regardless of the discipline or athletic level practiced [11,12,13]. Among professional athletes, O3I was assessed in endurance winter sports athletes, basketball players from the NBA-G-League, NFL football, NCAA football and rugby sevens athletes with mean values ranging from 4.29% to 5.02% [11,13,14,15,16]. Similarly, a lower-than-optimal O3I was observed in amateur athletes (endurance athletes, strength training amateurs or long-distance runners) with values of 5.40%, 4.58% and 5.70%, respectively [11,22,26]. The lowest values (4.13%) were observed in endurance athletes who were vegan [11] which may be explained by the lack of fish consumption in this population. The low O3I in athletes is consistent with the low O3I in the general population. In view of the confirmed low O3I due to the inadequate intake of EPA and DHA in athletes and the beneficial effects of these fatty acids (see below), athletes might wish to consider supplements that provide EPA and DHA.

## 5. EPA and DHA for Athletes’ Health

The impacts of EPA and DHA supplementation on health, which may indirectly improve exercise performance in athletes, include, but are not limited to, supporting cognitive function, neuroprotective effects and supporting recovery after injury [19,20,21,27]. In a study in amateur athletes, Fontani et al. observed that 5 weeks of supplementation with 1600 mg of EPA + 800 mg of DHA a day resulted in improvements in reaction time and cognitive performance, as well an enhanced state of vigour and a decrease in the negative mood state [19]. These results are consistent with studies conducted on female elite soccer players, in whom supplementation of 3500 mg of DHA a day over 4 weeks of training significantly improved complex reaction time and efficiency [20]. Moreover, adding 551 mg of EPA and the same amount of DHA to a protein-based supplement taken twice a day through 5 weeks during pre-season training resulted in a moderate reduction in fatigue in professional rugby players; however, the effects on sleep, stress and mood were modest [71]. Another area where EPA and DHA may have an impact is sport-related traumatic brain injury (TBI), the leading cause of brain injury in the world, which may affect athletes in a number of disciplines: combat sports, such as boxing or mixed martial arts, and team disciplines, such as football, rugby, horse riding and cycling [72,73,74]. It is estimated that yearly in the United States alone, 1.6–3.8 million incidents of sport-related TBI occur; therefore, its prevention seems to be of great importance in the context of athletes’ health and well-being [72]. Animal models have shown that supplementation with DHA, through activation of the Nrf2-ARE signalling pathway, can alleviate brain damage and promote recovery [75,76]. Preclinical models of brain injury indicate that EPA and DHA, especially DHA, confer neuroprotection and enhance recovery [77,78]. More recently, studies in human subjects exposed to repetitive head impacts have noted potential positive effects associated with EPA and DHA supplementation [21,79]. Studies by Oliver et al. and Heileson et al. evaluated how supplementation with EPA and DHA compared to placebo influences concentrations of serum neurofilament light (Nf-L), a biomarker of head trauma, which typically increases progressively over a season in American football players [21,79]. In the seminal study by Oliver and colleagues, supplementation with either 2 g, 4 g or 6 g of DHA per day for 189 days mitigated the increase in serum Nf-L over the course of a season irrespective of the application dose [21]. The period of supplementation coincided with periods when an elevated number and size of head impacts typically appear in American football athletes [21]. Similarly, Heileson et al. revealed that the administration of 2000 mg of DHA + 560 mg of EPA + 320 mg of docosapentaenoic acid (DPA) daily for 89 days attenuated Nf-L levels in American football athletes [79]. In contrast, Mullins et al. found that ~3.5 g/day DHA + EPA did not attenuate Nf-L levels after 17 or 26 weeks in collegiate American football players [80]. As noted by the authors, the sample size may have been too small to detect any differences. Most importantly, plasma DHA + EPA levels declined after 17 weeks and were similar to placebo by the end of the study, suggesting poor compliance. The authors found a moderately strong inverse correlation between the change in serum Nf-L and the change in %DHA + EPA. Finally, a subgroup analysis from Mullins and colleagues included neuroimaging that showed stronger structural and functional connectivity of the brain in American football players who supplemented with DHA + EPA compared to placebo, despite notable white matter damage [81]. Of note, Miller et al. recently determined that subjects supplied with 2000 mg DHA per day were symptom-free 5 days faster and were cleared for return to play 5.5 days faster compared to placebo following a diagnosed mTBI [82].

A promising field of investigation regarding the application of EPA and DHA in sport is their use during recovery from injury [23,24,25]. The first reports on this subject came from You et al., who fed rats a diet rich in corn oil or fish oil for 2 weeks and afterwards, while continuing this feeding, immobilized the animals’ hind limb for the next 10 days [23]. Measurements of the soleus muscle mass taken before and at the end of the experiment showed less atrophy-induced decline of this muscle in animals fed a diet rich in fish oil. In addition, in rats consuming fish oil, disturbances in the activation of proteins (Akt and p70 S6 kinase) that are involved in muscle protein synthesis and the expression of genes associated with muscular atrophy were less than in rats on the high corn-oil diet [23]. However, in a follow-up study, You et al. revealed that continuation of the use of fish oil during the phases of reimmobilization and immobilization through 13 and 10 days, respectively, have a negative impact on muscle recovery through the inhibition of increases in Akt and p70 S6 kinase activation and synthesis of PGF2α [24]. Using an intermittent arm-immobilization model (9 h/d), Bostock et al. found that EPA + DHA supplementation in a dose of 2.16 g a day for two weeks may mitigate skeletal muscle atrophy in young adults and reduce subcutaneous adipose tissue accumulation during periods of disuse [83]. In an elegant experiment, McGlory et al. performed two-week unilateral leg immobilization in physically active young women. They found that supplementation with 2.97 g of EPA and 2.03 g of DHA a day, starting 4-weeks prior to immobilization and throughout its duration, attenuated the declines in skeletal muscle volume and mass which later completely recovered to preimmobilization values with no such results seen in the placebo group [25]. This confirms previous studies indicating that EPA and DHA supplementation can overcome the phenomenon of anabolic resistance, i.e., limited muscle protein synthesis (MPS) [55]. Although more research is needed in this area, particularly with male athletes, it appears that the application of EPA and DHA may be an effective strategy for athletes undergoing a period of recovery from injury.

## 6. EPA and DHA and Recovery from Exercise

There has been considerable research interest in EPA and DHA as dietary support for post-exercise recovery. Much of the attention comes from the aforementioned evidence supporting a role in promoting muscle remodelling and repair via membrane incorporation, anti-inflammation and immune surveillance [29,30]; the resolution of inflammation could also be important here. One of the most common methods to study the relationship between supplementation and recovery is to induce muscle damage through near-maximal or maximal eccentric contractions, e.g., downhill running. Muscle damage typically manifests via a range of symptoms to include delayed onset muscle damage, a reduced range of motion, muscle swelling and a loss of force production [84]. The association between exercise-induced muscle damage and EPA and DHA supplementation has been fairly well researched [27,85,86]. In doses varying from 0.54 to 4.20 g EPA + DHA administered daily for between 7 and 70 days, EPA and DHA principally reduce muscle soreness regardless of dose, duration or the muscle-damage model employed [27,71,87,88,89,90,91,92,93,94,95]. In a field-based study in rugby union players, Black et al. observed that EPA + DHA supplementation reduced fatigue after 20 days and reduced muscle soreness that coincided with an improved jump performance after 35 days, despite the rigors of pre-season training [71]. In a 4-week trial, 2.7 g EPA + DHA per day significantly reduced muscle soreness and pain compared to placebo at 72 and 96 h following an upper body damage protocol [92]. Jouris et al. found that, compared to placebo, seven days of DHA supplementation (3 g/d) significantly reduced muscle soreness at 48 h when weight was applied to the arm and during extension after an upper-body muscle damage protocol [91]. Unlike muscle soreness, the preservation of power and strength may be dose-dependent. For example, Van Dusseldorp et al. provided participants with 1.40, 2.80 or 4.20 g EPA + DHA per day for about 52 days before and during exercise-induced muscle damage [93]. Interestingly, the highest-dose group experienced the fastest recovery in jump height (1 h) and strength (72 h). Despite the debated heterogeneity between studies [96], EPA and DHA supplementation reduces subjective muscle soreness and improves some aspects of physical performance, such as the countermovement or vertical jump, which may be advantageous to athletes in any sporting context.

## 7. EPA and DHA and Training Adaptation

Recent human studies suggest enhanced training-induced adaptations as a result of EPA + DHA supplementation [22,26,97]. In a study by Heileson et al. in recreational exercisers, RET-induced adaptations were evaluated after a 10-week RET program taken three times a week with supplementation of 2.275 g of EPA + 1.575 g of DHA daily or safflower oil at 4.5 g a day. Absolute and relative increases in the 1-repetition maximum in a bench press (1RMBP) and relative increases in the 1-repetition maximum in a squat (1RMSQT) were observed in subjects taking EPA + DHA compared with placebo, which suggests enhanced RET-induced adaptations in these participants [22]. Interestingly, contrary to the authors’ assumptions, the protocol did not contribute to an increase in lean body mass (LBM), which is a commonly observed effect in similar studies conducted on healthy adults. As assumed by the authors, a fibre type transformation, reduction in intramuscular fat and increased neuromuscular activation may provide an explanation for improved muscle strength measurements [22]. Moreover, in a 6-week study in resistance-trained men, Philpott et al. evaluated the influence of 2 g of EPA and 2 g of DHA per day compared to a placebo on changes in body composition and muscular strength over a short weight loss program achieved via 40% calorie restriction [97]. At the end of the experiment, in the EPA + DHA group, the non-dominant 1RM leg extension was significantly higher than at baseline, despite significant weight loss. As in the previous study, contrary to the authors’ hypothesis, no attenuation of LBM decline was observed in subjects using EPA + DHA [97]. The results of this study may be valuable for athletes who want to reduce body weight without compromising training effects [97]. EPA and DHA have also been shown to affect oxygen kinetics: cycling efficiency or peak/maximal oxygen uptake which, however, do not translate into improved physical performance as such [26,98,99,100]. In our recent 12-week study, amateur runners underwent progressive, controlled endurance training four times a week with supplementation of 2234 mg of EPA and 916 mg of DHA daily (OMEGA group) or 4000 mg of medium-chain triglycerides (MCT group) as a placebo. Similar to earlier studies, improvements in running economy were observed at the end of the 12-week period only in runners using EPA + DHA. Moreover, despite the lack of differences between runners from both groups in VO_2_peak at the end of the experiment, out of 14 participants in the OMEGA group, 13 showed an enhancement in VO_2_peak compared with the MCT group, in which only 9 out of 12 participants showed enhancement [26]. Improved adaptation to endurance training in response to EPA + DHA supplementation may explain these results, although more studies are needed in this area to draw clear conclusions [26]. Importantly, many authors point to the paucity of long-term studies using adequate dosage of EPA and DHA and other limiting factors, such as the lack of homogeneous low O3I levels as an inclusion criterion for participants, in obtaining a full picture of effectiveness of EPA and DHA [2,22,96,101]. Following supplementation with a dose of 3.27 g of EPA + DHA per day for 4 days per wk, the time required to achieve peak incorporation of EPA and DHA into erythrocyte membranes which reflects their concentration in target tissues, was estimated at 55 and 136 d for EPA and DHA, respectively [102]. However, the exact duration needed for the maximum incorporation of EPA and DHA into muscle cell membranes is unknown [25], and this would allow us to establish a causal relationship between EPA and DHA supplementation and changes in muscle performance parameters.

## 8. Potential Adverse Impacts of EPA and DHA Supplementation

Whilst focusing on the beneficial impacts of EPA and DHA and considering their utility in the athlete, it is important to also consider any adverse impacts. Among the most frequently discussed are gastrointestinal (GI) disturbances, altered platelet function and consequently the increased risk of bleeding and questions about lipid peroxidation.

GI disturbances, particularly fishy aftertaste and fishy “burps”, are often reported for some individuals in trials of supplemental EPA and DHA. A meta-analysis of 10 randomized controlled trials of EPA and DHA either alone or in combination (doses up to 1.86 g EPA and/or DHA/day and durations up to 52 weeks) identified that no serious adverse events had occurred, that there was no difference in adverse events between EPA + DHA and placebo and that GI disturbances were the most common adverse event reported [103]. There was no difference in the percentage of participants reporting GI disturbances with EPA + DHA compared with placebo. It is noteworthy that this analysis did not include studies using EPA + DHA doses of 2 g/day or more. A more recent meta-analysis of 21 randomized controlled trials of prescription-grade EPA + DHA, providing between 1.8 and 4 g EPA + DHA/day for between 6 weeks and 5 years, identified no evidence of serious adverse events but an increase in fishy taste, fishy “burps” and nausea with combinations of EPA and DHA compared with placebo [104]. These possibilities are something those contemplating using EPA and DHA supplements should be aware of. On the other hand, millions of people regularly use such supplements without complaint.

The starting point for discussion about EPA, DHA and bleeding time is the early studies conducted in the Greenland Inuit in whom such a phenomenon was observed [105]; daily intake of EPA + DHA in that adult population was estimated to be an average of 15 g/day [106]. The effect on bleeding was related to changes in production of pro- and anti-thrombotic oxylipins from arachidonic acid and EPA (thromboxanes and prostacyclins). However, clinical studies, in which the parameters involved in blood coagulation and bleeding time itself were assessed, did not confirm the effects on coagulation or bleeding time, even when high doses of EPA + DHA were used [107,108]. One fairly recent study was conducted with the aim of directly testing the effect of high-dose EPA + DHA on blood loss at surgery [109]. Over 1500 patients who were due to undergo cardiac surgery were randomized to receive a placebo or high doses of EPA + DHA prior to (up to 10 g/day) and following (2 g/day) surgery. EPA + DHA did not increase blood loss: there was no increase in bleeding (in fact, there was a trend to less blood loss) and a significant reduction in the need for transfusion with high-dose EPA + DHA, and a higher DHA status was associated with less blood loss [109]. Given these findings, potential concerns about bleeding with a standard low-to-moderate dose EPA + DHA supplementation seem unwarranted.

Concerns about lipid peroxidation arise due to the nature of the polyunsaturated bonds in EPA and DHA, which, relative to saturated bonds, are more easily oxidized, resulting in the production of fatty acid free-radicals (lipid peroxides) which are harmful to cellular integrity [110]. This effect can be mitigated by including a sufficient antioxidant in the oil, by keeping supplements stored in cool, dark places and by using supplements before their expiry date. The European Food Safety Authority (EFSA) noted that intakes of EPA and DHA either alone or in combination at doses up to 5 g/day for 16 weeks do not induce changes in lipid peroxidation [111]. Lien [112] reviewed the safety of DHA using data from studies in rodents and humans. The conclusion was that ‘’DHA supplementation studies in adults have employed doses ranging from less than 1 to 7.5 g/d and have not resulted in any consistent adverse responses in platelet function, lipid levels, in vivo oxidation parameters, glycemic control, or immune function. In conclusion, DHA consumption does not result in consistent adverse events in infants or adults”. Moreover, the EFSA has made several statements confirming the lack of negative health consequences of EPA and DHA supplementation [111], including:

- “long-term supplemental intakes of EPA and DHA combined up to about 5 g/day do not increase the risk of spontaneous bleeding episodes or bleeding complications even in subjects at high risk of bleeding”;
- “supplemental intakes of EPA and DHA consumed either alone or in combination at doses up to about 5 g/day for up to 16 weeks do not induce changes in lipid peroxidation which might raise concern in relation to cardiovascular disease risk as long as the oxidative stability of these [fatty acids] is guaranteed”;
- “supplemental intakes of EPA and DHA combined at doses up to 5 g/day, and supplemental intakes of EPA alone up to 1.8 g/day, do not raise safety concerns for the adult population”;
- “supplemental intakes of DHA alone up to about 1 g/day do not raise safety concerns for the general population”.

In the US, the FDA has stated that EPA and DHA are “generally regarded as safe” up to an intake of 3 g/day [113]. The American Heart Association advises 2 to 4 g EPA + DHA per day for triglyceride lowering [114].

## 9. Other Points to Consider

Translating the large body of human research around EPA and DHA into recommendations for athletes, beyond the recommendations that already exist for the general population, raises a number of questions. These relate to dose, duration (timing), chemical formulation and relative amount of EPA vs DHA.

**Figure 2:**
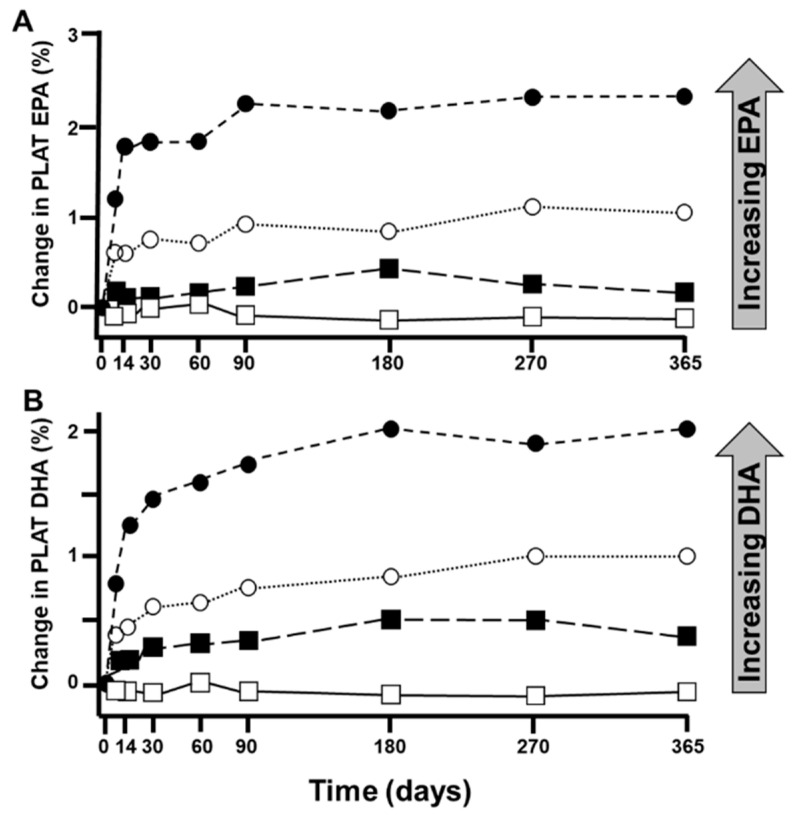
Time course of changes in (**A**) eicosapentaenoic acid (EPA) and (**B**) docosahexaenoic acid (DHA) content of human platelets (PLAT) in participants consuming placebo oil or one of three doses of EPA + DHA. Healthy participants supplemented their diet with capsules providing 0 (open squares), 3.27 (closed squares), 6.54 (open circles) or 13.08 (closed circles g EPA + DHA per week for a period of 12 months; the ratio of EPA to DHA was 1:1.1. Platelets were isolated from blood at 0, 1, 2, 4, 8, 12, 24, 36 and 52 weeks and the fatty acid composition determined by gas chromatography. Data are the mean change from time 0 from at least 30 participants per group. Taken from the American Journal of Clinical Nutrition, Vol 96, LM Browning et al., Incorporation of eicosapentaenoic and docosahexaenoic acids into lipid pools when given as supplements providing doses equivalent to typical intakes of oily fish, Pages 748–758, Copyright (2012), with permission from the American Society of Nutrition [102].

The biological effects, physiological outcomes and health impacts of EPA and DHA are dose-dependent, although they likely plateau once a particular dose is reached and different outcomes show different dose responses [115]. This relates to the mechanism by which the biological effect, physiological outcome or health impact is influenced by EPA and DHA and the sensitivity of that mechanism to a particular exposure to the fatty acid. Detailed human studies of blood pools such as blood lipids, erythrocytes, leucocytes and platelets identify clear linear dose-response relationships of incorporation of both EPA and DHA that saturate after a period of time [102,116,117,118,119] (Figure 2). It is likely that such dose-response relationships occur in tissues such as skeletal and cardiac muscle, although these are not described in humans.

**Figure 3:**
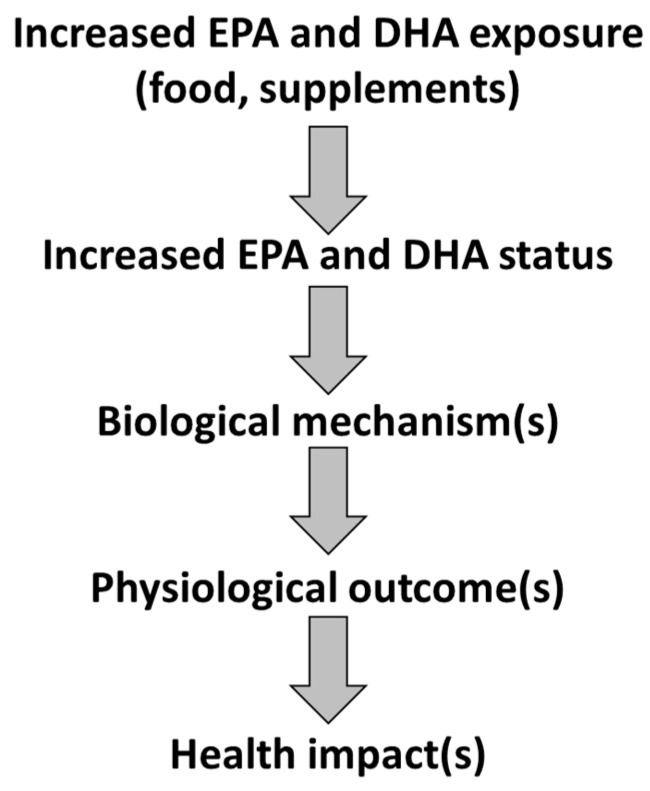
Causal chain linking increased intake of EPA and DHA from food (e.g., fatty fish) or from supplements to physiological outcomes and health impacts.

This creates a link between exposure and outcome as shown in Figure 3.

Because different biological effects and health outcomes follow different responses to any given dose of EPA and DHA, there is no single dose that will be effective for all outcomes; some outcomes will be sensitive to an intake of a few hundred milligrams of EPA + DHA each day, while other outcomes may require 1, 2 or even more grams per day [115].

**Figure 4:**
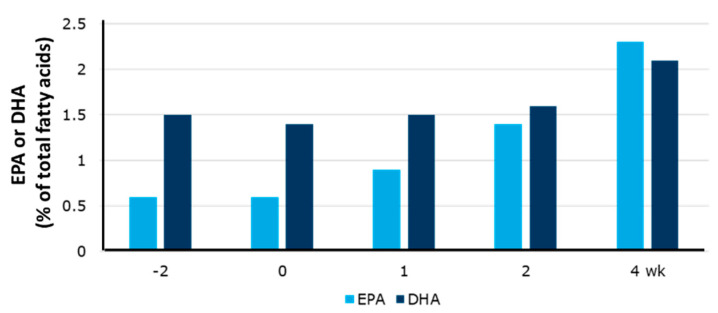
Time course of the eicosapentaenoic acid (EPA; light blue bars) and docosahexaenoic acid (DHA; dark blue bars) content of human skeletal muscle in participants consuming EPA + DHA. Healthy young males supplemented their diet with capsules providing 3.5 g EPA + 0.9 g DHA per day for a period of 4 weeks. Muscle (*Vastus lateralis*) biopsies were collected at −2, 0, 1, 2 and 4 weeks and the fatty acid composition determined by gas chromatography. Data are the mean content of EPA and DHA (% of total fatty acids) for 10 participants and are taken from McGlory et al. [124].

A second question is timing. Once again, detailed human studies of blood pools such as blood lipids, erythrocytes, leucocytes and platelets identify clear time-dependent relationships of incorporation of both EPA and DHA that eventually saturate [119,120,121,122,123] (Figure 2). Generally speaking, incorporation is fastest into the most rapidly turning over pools; thus, it is faster into platelets and leukocytes than into erythrocytes [102]. Leukocytes are saturated within a matter of about 4 weeks [119,121], while erythrocytes require 4 to 6 months [102,122]. It is not clear how long it takes for human tissues to become saturated with EPA and DHA. It is commonly observed that EPA is incorporated more quickly than DHA: this is well described for blood lipids, blood cells (platelets (Figure 2), leucocytes, erythrocytes) and tissues where time courses have been studied in humans such as skeletal muscle ([124]; Figure 4). The new steady-state level of EPA and DHA is maintained so long as the intake of the fatty acid remains unchanged. However, if the intake is decreased (e.g., a person stops taking the supplement), EPA and DHA are lost over time: this has been described for blood lipids, platelets, leukocytes and erythrocytes [116,121,122]. Just as DHA is incorporated more slowly than EPA, it is lost more slowly [116,121,122]. In most cases, individuals will eventually lose the benefits of EPA and DHA once the fatty acids are lost and the status returns to what it was prior to increasing the intake of EPA and DHA.

EPA and DHA are available in different chemical forms in foods and in supplements. In fish, in most fish oil type supplements and in “concentrates”, EPA and DHA are mainly found in triglycerides. However, phospholipid forms are available; for example, in krill oil, some EPA and DHA is present in phospholipids and some in triglycerides. There are also highly-concentrated pharmaceutical-grade preparations that are composed of ethyl esters of both EPA and DHA, ethyl esters of EPA or free EPA and DHA. When triglycerides, phospholipids and ethyl esters are consumed, they need to be hydrolysed (“digested”) in the small intestine to free the EPA and DHA for absorption. This process is promoted by a meal, particularly one that contains fat. Thus, in people with normal GI physiology, taking supplements with or around the same time as a meal allows for the efficient digestion and absorption of EPA and DHA irrespective of its chemical form [125]. However, taking supplements away from a meal (e.g., on an “empty stomach”) could significantly impact EPA and DHA bioavailability. This is clearly shown in trials with ethyl esters of EPA and DHA, where intake in the absence of food was not followed by the appearance of EPA and DHA in the bloodstream [126,127]. This is not an issue with free EPA and DHA since digestion is not required to enable absorption [128] or with formulations that can “self-emulsify” [129,130,131]. It has been argued that phospholipids might enhance bioavailability [132]. Schuchardt and Hahn [133] reviewed the literature on EPA and DHA bioavailability from different chemical formulations and found it to be inconsistent, perhaps because studies had not always carefully matched the EPA and DHA contents of the different formulations being compared. Advice to athletes or others would be to take supplements of EPA and DHA with meals in order to maximise the bioavailability of the fatty acids.

The final question is which is more important: EPA or DHA? Again, the answer may differ according to the biological system of interest. For example, specific parts of the eye and brain are rich in DHA, and DHA is vital to visual and cognitive function. EPA does not have those same effects and EPA is not able to replace DHA for visual and cognitive development and function. On the other hand, both EPA and DHA lower triglycerides, decrease blood pressure and improve vascular function [134]. Both EPA and DHA are anti-inflammatory and both give rise to SPMs [41,42,43]. Thus, qualitatively, the effects of EPA and DHA on many biological outcomes are similar. Compared to the number of human trials of EPA and DHA combined, there are very few studies directly comparing near-pure EPA with near-pure DHA; however, using data from the trials that had been published up to 2017, a systematic review identified that DHA was more effective than EPA at improving some, but not all, cardiovascular risk factors [134]. Fish and most supplements contain both EPA and DHA and recommendations for EPA and DHA intake for the general population do not distinguish between the two fatty acids, although recommendations for pregnant and breastfeeding women emphasise the need for DHA [62,63]. Advice for supplement selection would be to look for a high content of EPA or DHA or both, because there is insufficient information to indicate that either EPA or DHA is superior for any specific desired outcome (beyond visual and cognitive development and function in early life).

## 10. Conclusions

The most biologically active *n*-3 PUFAs EPA and DHA are present in high amounts only in oily fish and selected species of algae. EPA and DHA have multiple actions, which are mainly related to improved physiology and health benefits. Consequently, there are many recommendations for the general population to consume EPA and DHA, preferably from fish. Supplements are also a source of EPA and DHA. EPA and DHA consumption seems to be insufficient in the athlete population according to the accepted status marker O3I. Considering their broad spectrum of actions, including but not limited to supporting nervous system function, maintaining muscle mass after injury and improving training adaptations and the lack of adverse effects at dosing regimens that might be recommended (e.g., 2 g/day), it seems reasonable for athletes to consider using EPA and DHA supplements. These would also benefit their long-term health. Nevertheless, more research is needed in athletes including a better exploration of effective doses and durations. In addition, more consistent effects on outcomes of relevance to athletes are required; obtaining this evidence is related to the aforementioned questions of dose and duration. Given these uncertainties, it is reasonable for EPA and DHA to be placed in the AIS category B of sports supplements. However, more and better research, enhancing the evidence base and removing uncertainties may lead to them being placed in category A in the future.
